# Assessment of care provision for hypertension at the emergency Department of an Urban Hospital in Mozambique

**DOI:** 10.1186/s12913-019-4820-8

**Published:** 2019-12-18

**Authors:** Neusa BAY, Edna JUGA, Carlos MACUACUA, José JOÃO, Maria COSTA, Simon STEWART, Ana MOCUMBI

**Affiliations:** 1 Mozambique Institute for Health Education and Research, Cidade de Maputo, Mozambique; 2 Instituto Nacional de Saúde, Vila de Marracuene, Estrada Nacional N°1, Parcela N°3943, Província de Maputo, Mozambique; 3 Hospital Geral de Mavalane, Cidade de Maputo, Mozambique; 4 Torrens University, Adelaide, Australia; 5 Universidade Eduardo Mondlane, Cidade de Maputo, Mozambique

**Keywords:** Systemic hypertension, Management Cascade, Medicine availability, Affordability

## Abstract

**Background:**

Management of hypertension in Mozambique is poor, and rates of control are amongst the lowest in the world. Health system related factors contribute at least partially to this situation, particularly in settings where there is scarcity of resources to address the double burden of infectious and non-communicable diseases. This study aimed to assess the management of hypertension in an emergency department (ED).

**Methods:**

During a pragmatic and prospective 30-day snapshot study (with 24 h surveillance) and random profiling of one-in-five presentations to the ED of Hospital Geral de Mavalane, Maputo, we assessed patient’s flow and care, as well as health facility’s infrastructure and resources through direct observation. Reports from pharmacy and laboratory stocks were used to assess availability of diagnostics and medicines needed for hypertension management.

**Results:**

**The** 1911 hypertensive patients included in the study had several stops during their journey inside the health facility and followed a non-standardized care flow. No clinical protocols or algorithms for risk stratification of hypertension were available. Stock-outs of basic diagnostic tools for risk stratification and medicines were registered. The availability of medicines was 28% on average.

**Conclusions:**

Critical gaps in health facility readiness to address arterial hypertension seen in ED were uncovered, including lack of clinical protocols, insufficient availability of diagnostics and essential medicines, as well as low affordability of the families to guaranty continuum of care. Innovative financing mechanisms are needed to support the health system to address hypertension.

## Background

Hypertension is the leading risk factor for premature death and disability and constitutes an important cause of health services demand worldwide [1]. Global disparities in its prevalence and awareness have been increasing [2**,** 3].

In Mozambique, hypertension prevalence is among the highest in developing countries, and has increased in people aged 25–64 from 33.1 in 2005 to 38.9% in 2015 (p 0.048) with low awareness (14.5%), treatment among those aware (50.1%) and control among the treated (44.5%) [1**,** 4]. Hypertension is an important driver of heart failure and stroke in this country [5**,** 6] and thus constitutes a priority in Mozambique’s strategic plan for non-communicable diseases (NCD) prevention and control [7]. However, the national health information system still does not systematically perform surveillance of hypertension and its complications.

Initial surveillance data on NCD targeted by the World Health Organization (WHO) global strategy for the prevention and control of NCD showed prominence of hypertension as a cause of poor outcomes [8]. Due to the continuing need for major efforts to control infectious endemic diseases in Mozambique, the burden imposed by NCD constitutes a high risk for the sustainability of the health system. Moreover, because no funding mechanisms are available to sustain national NCD program’s activities within the National Health Service (NHS) in Mozambique, insufficient investments have been made on health facilities to address this double burden of disease. Our study aimed at describing care provision for hypertensive patients assisted at the emergency department (ED) of a first-referral urban hospital, often an entry point for patients into the NHS.

## Methods

The National Health System in Mozambique comprises the public sector, the for-profit private sector, the non-profit private sector and the community sector. The public sector, which is the National Health Service (NHS), is the main provider of health services on a national scale, and is organized into four levels of care (primary - health centres; secondary – district hospitals and general urban hospitals; tertiary – provincial hospitals; quaternary – specialized and sub-specialty hospitals). Treatment and diagnostic resources for the NHS depends on a supply chain structured in 5 levels. The *Central Level* with three regional warehouses for Southern, Central and Northern Region supplying central and general hospitals and provincial deposits; *the Provincial Level* with provincial deposits supplying tertiary and lower level facilities, including some rural hospitals and district health centers; the *District Level* deposits supplying all health facilities in one district. Pharmacies in health centers and community Health Workers - supplied by the nearest district deposits or health centers – constitute the primary and community levels, respectively [9]. Levels of prescription have been defined to ensure that each health professional prescribes according to their training background and the health facility level [10]. Care cost is $0.02 (1MZN) per appointment, nil for complementary exams and $0.08 (5MZN) for each prescription with one or more medicines. The total amount charged corresponds to 0.14% of the patient’s salary, considering the official minimum wage [11].

### Setting

We performed an assessment of hypertension care in patients assisted around the clock at *a* first-referral hospital in Mozambique’s capital city - *Hospital Geral de Mavalane* (HGM). This is an urban secondary level 265-bed hospital serving a population of nearly 800,000; it is a pilot sentinel site for NCD without patient electronic management system, and where hospital identification cards and medical files are used for patients with chronic condition.

### Sampling

During the pilot-phase of the MOZambique snApshot of emeRging Trends (MOZART) Disease Surveillance Study cohort [12], with a target of at least 1000 patients per health facility studied, and based on previous knowledge of the caseload of patients in the hospital a ratio of selected presentations of 5 to 1 was established to achieve this minimum target. Over 30 consecutive days with 24-h coverage patients of any age presenting to the emergency department of the HGM were registered and randomly selected for a more detailed profiling. Selected participants were approached by trained staff for informed consent; the first patient to be selected during each shift was selected randomly (from a list of the first five patients assisted); then, every fifth patient would be invited to participate. If a randomly selected patient was unable to respond to the nurses or the study survey team (i.e., unconscious, confused due to mental illness, severely ill, or under the influence of illicit drugs) informed written consent, demographics and risk factors data (including arterial hypertension), were obtained from a family member or spouse (legal guardians) by a medically qualified researcher. Even if the patient was not included in the study, he/she would be registered through the usual health information system, and therefore included in the full count of patient activity during the 30-day snapshot period.

### Data collection

For each patient presenting at the ED pre-designated data collection was performed by trained personnel, with skills for health surveillance and/or medical and nursing qualifications, using electronic capture. Direct observation was done using several trained health workers in each station and registered in paper-based case report forms (CRF) for full registry of the patient’s profile (name, residential suburb, contact details and hospital identification, demographics, risk factor’s data, medical history, clinical data, management information, immediate outcomes and time of discharge); researchers from INS entered data in electronic CRF on dedicated study laptops with restricted access ensuring the anonymity of each patient. Those patients admitted to wards or transferred to an elective unit were followed-up through their hospital records for the later completion of management and outcome data. At the completion of data collection, each case was allocated a final ICD 10 diagnosis (a web-link providing access to all ICD-10 coding for reference is provided). Participants were given a unique identifier to protect their anonymity and only de-identified data were used to generate study findings. Elevated BP was defined by systolic ≥140 mmHg and/or diastolic BP ≥90 mmHg (Grade I) - for adolescents above 15 years and adults (≥20 years); grade II hypertension 160–179 and/or 100–109 and grade III ≥ 180 and/or ≥ 110 [13].

To describe the management of hypertension we used direct observation of patient’s flow during the study, including collection of data on the infrastructure and human resources. Using the Package of Essential NCD interventions for primary health care in low-resource settings (PEN) [14] we selected diagnostic tools and medicines that are needed for hypertension management; we then consulted the reports from pharmacy and laboratory stocks to assess availability of diagnostics and medicines. The WHO essential medicines target of 80% was used to define availability. To define affordability the patient monthly capacity to purchase a medicine, we considered the national minimum wage and the WHO/HAI external reference pricing (available at the WHO/HAI database of medicine prices, availability, affordability and price components) [15] to calculate the number of work days, as well as the percentage of the monthly minimum wage that a patient needs to obtain the medicines prescribed.

#### Data analysis

Data was analysed using descriptive statistics (percentages and proportions); patient’s flow in the health facility and availability/affordability are presented graphically.

#### Ethical issues

We have followed the STROBE guidelines for the conduct and reporting of observational studies [16]. The study had ethical approval by the National Bioethics Committee in Mozambique (IRB 0002657). Informed consent was obtained from all patients participating in the MOZART study.

## Results

### Hypertension patients profile

Out of the 8890 patients assisted during the study period we selected 1911 cases, of which 14 refused to participate and 55 were lost to follow up. For 197 patients data was incomplete. Among the remaining 1645 patients, 556 were children under 10 who were excluded from analysis. Of the remaining 1089 patients, 28 patients did not have their blood pressure measured and thus were excluded from analysis and 47 had the diagnosis of hypertension but the attending health worker had not registered the blood pressure values. Thus, 1014 patients were included in the analysis - 574 (56.6%) females, 263 (16%) adolescents (10–19 years) - of which 322 had high blood pressure; among these, 26 (2.6%) were adolescents > 15 years. Of the 322 hypertensive patients (females 168, 52.2%), 68 presented with systolic blood pressure ​​ ≥ 160 mmHg and/or diastolic blood pressure ≥ 100 mmHg. From these hypertensive patients 37 were transferred to the hospital’s intensive care unit, 6 (2%) were admitted to hospital wards, and 3 (1%) needed transfer for specialized care in referral hospitals due to acute complications.

### Patient flow

**Fig. 1:**
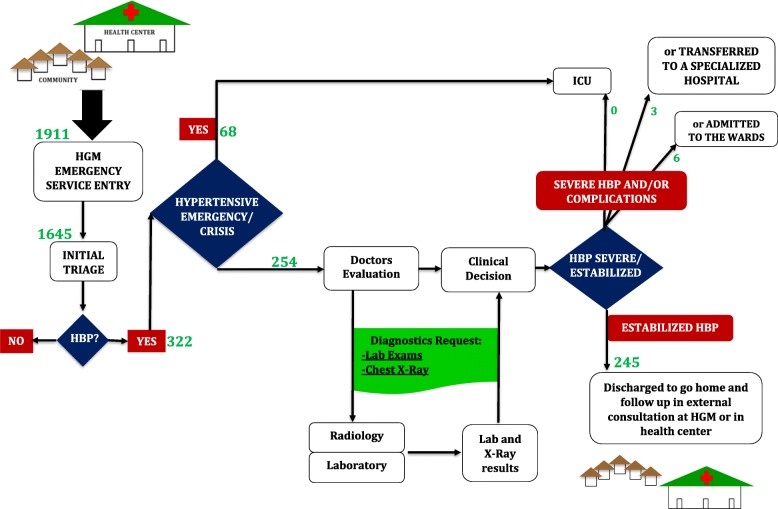
Flow of Patients. Describes the flow of the patient with hypertension from arrival at the ED to discharge, indicating the number of hypertensive patients that were seen during the MOZART study

Hypertensive patients had several stops during their journey inside the health facility. After initial triage at entry they could be sent directly to the ICU in case of hypertensive emergency/crisis. All other cases would wait for doctor’s evaluation, blood collection for biological profiling at the laboratory, eventually performing chest X-ray and, finally, returning the results to the assisting doctor, who would decide if they were sent to the ICU, transferred to a specialized hospital, admitted to the wards or discharged. (Fig. 1).

### Facility resources

**Fig. 2:**
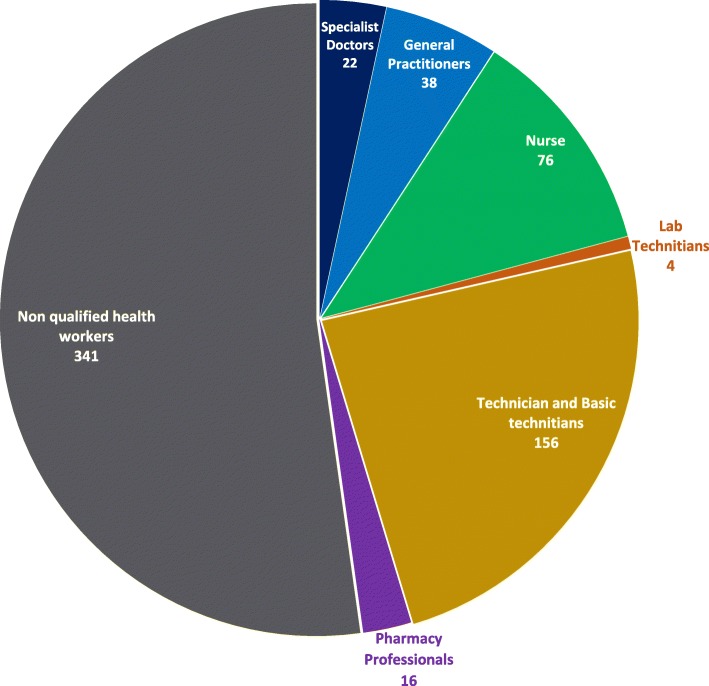
Human Resources Availability. The distribution of health professionals involved in patient care and administrative roles in this health facility is presented, including personnel allocated to NCD complementary services. The numbers are shown per type of services needed

Basic clinical equipment for diagnosis was available (sphygmomanometers, scales/altimeters, oxymeters, electrocardiograph, X-ray machine and ultrasound). However, no rapid tests were available (urine, blood glucose or HbA1c) for prompt risk stratification, and occasional stock-outs were registered for cholesterol and uric acid. Regarding human resources availability, the hospital had 38 general practitioners working around the clock at the ED who assisted patients and took decisions on: admitting them to the intensive care unit or transfer them to a referral hospital; admitting them into the hospital wards; sending them to book appointments for outpatient clinic for follow up with specialists within the hospital; or send them to a satellite health centers and the ED. There was no variation in availability of clinicians and other personal during the 24 h. The staff of the whole hospital is presented in Fig. 2. No guidelines for risk stratification of hypertension were available in the consultation rooms, nor were manuals or algorithms.

### Availability & Affordability of medicines

**Fig. 3:**
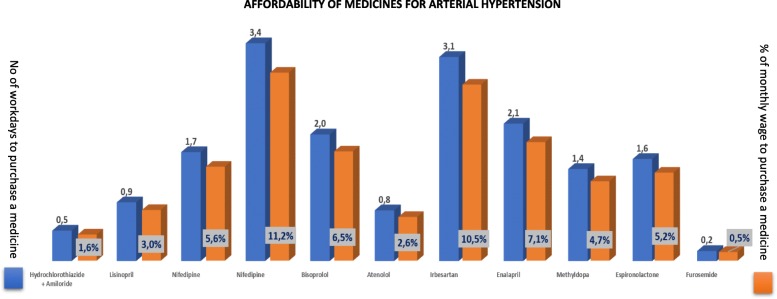
Work days & Affordability of Medicines. The medicines selected corresponded to the different lines of treatment for arterial hypertension and its complications prescribed at a first-referral hospital according to the national medicines formulary. The graph presents data on affordability of anti-hypertensive drugs (when obtained outside the health facility)

The availability of medicines was 28% on average; 3 to 6% for calcium channel blockers (amlodipine) and aldosterone antagonist (spironolactone), respectively; between 51 to 70% for adrenergic modifier/alpha-2 agonist (methyldopa), loop diuretic (furosemide) and angiotensin converting enzyme inhibitor (enalapril); only nifedipine 30 mg was available in reaching the WHO essential medicines availability target of 80% (availability 145%). Beta-blockers, angiotensin-II receptors antagonists, angiotensin converting enzyme inhibitor (lisinopril) and the combination (Hydrochlorothiazide and Amiloride) had 0% availability during the study period (Table 1). For medicines not available at the health facility pharmacy patients would spend from 0,5–11.2% of their monthly wage to purchase a single medicine, corresponding to 0,2–3,4 work days. The most affordable medicines found in this context were Hydrochlorothiazide, Methyldopa, Atenolol and Furosemide. The later - used to treat hypertension complications - was the most affordable medicine, while Nifedipine corresponded to 11.2% of the monthly minimum wage, approximately 3.4 work days. (Fig. 3**).**

**Table 1:** Hypertension Medicines Availability. Drugs and formulations prescribed to patients with hypertension, its estimated need and availability in the hospital

Therapeutic Group	Generic Name / International Nonproprietary Name	Pharmaceutical form	Strength	Posology	Estimated need for one month	% of medicines available	SDG EM Target Availability
Thiazide diuretic + potassium sparing diuretic	Hydrochlorothiazide Amiloride	Tablets	50 mg + mg	0,5	7905	0%	< 80%
Angiotensin Converting Enzyme Inhibitor	Lisinopril	Tablets	20 mg	1	15,810	0%	< 80%
Calcium Channel Antagonists	Nifedipine	Tablets	30 mg	1	15,810	145%	**> 80%**
Calcium Channel Antagonists	Nifedipine	Tablets	60 mg	1	15,810	0%	< 80%
Beta blocker	Bisoprolol	Tablets	5 mg	1	15,810	0%	< 80%
Beta blocker	Atenolol	Tablets	50 mg	0,5	7905	0%	< 80%
Angiotensin-II Receptor Antagonist	Irbesartan	Tablets	300 mg	1	15,810	0%	< 80%
Angiotensin Converting Enzyme Inhibitor	Enalapril	Tablets	20 mg	1	15,810	60%	< 80%
Adrenergic modifier / alpha 2 agonist	Methyldopa	Tablets	250 mg	1	15,810	51%	< 80%
Aldosterone antagonist	Spironolactone	Tablets	25 mg	1	15,810	6%	< 80%
Loop diuretic	Furosemide	Tablets	40 mg	1	15,810	70%	< 80%

Obs: The availability was calculated for all patients (527) reported to have hypertension assisted in October 2017 (considering that this number includes the patients transferred from the ED, and medication should be available for all of them). SDG EM: Sustainable Development Goals Essential Medicines

## Discussion

This study unveils a high occurrence of severe hypertension in patients assisted at the emergency department of this first urban hospital, in a context of health facility barriers to good quality of care for hypertensive patients, particularly i) the lack of availability of guidelines and clinical protocols for management for use by health professionals; ii) the absence of standardized risk stratification, follow up and referral; iii) deficient strategies to ensure continuum of care; and iv) low availability of key consumables for laboratorial diagnosis and medicines. These organizational weaknesses increase the risk of poor outcomes, particularly in patients already with complications, or in those who have grade severe hypertension and/or associated risk factors.

Recently, a clinical study from the same health facility reported on poor short-term outcomes of severe and complicated hypertension [17]. In this report, young adults with high-risk profile and multimorbidity had high case-fatality, occurrence of established and/or de novo target organ damage, and hospitalisation rates on six-month follow-up. Additionally, this hospital cohort had low control rates and a concerning pattern of multimorbidity (more than two co-existing chronic conditions) that significantly increase the pressure on already stretched health systems.

Mozambique’s low rates of medical doctors and trained specialists [18**,** 19] - as well as nurses and allied professionals - are among the lowest in the world. To address the lack of workforce to tackle endemic infectious diseases (such as malaria, tuberculosis, HIV/AIDS and neglected parasitic diseases) the NHS has accommodated health professionals with basic- and mid-level training who are trained for triage and management of simple cases, using algorithms for diagnosis, management and referral [20] Most of these clinical guidelines are recommended or endorsed by the WHO, and are easily incorporated in the health systems due to being largely subsidized by international funding organizations. In contrast, for NCD clinical protocols and management algorithms are still not consensual, and rarely used in our setting, despite their known role in improving the quality of care. While recognizing that the number of health professionals is reduced, lack of risk-based management, unavailability of standard evidence-based clinical protocols and low access to essential anti-hypertensive drugs [21] – all essential to ensure high quality of care and prevent complications - are major gaps in hypertension care. Addressing these gaps would allow standardization of care provided by front-line health workers and probably improve outcomes in secondary level health facilities. Indeed, the fact that hypertension-related complications have become more diagnosed at secondary and tertiary care levels in some settings in Africa, is probably indicative of poor management of hypertensive patients at primary health care level.

Because ED in low-income settings are often the first contact point of patients with the health system, strategies to reduce lost opportunities for early diagnosis, prompt management and secondary prevention are needed. ED may be the only or most used form of contact with the health system for certain population groups in our health system, particularly adolescents and men who are not covered by the strong maternal and child health care programs. Owing to the high prevalence of hypertension in Mozambique [4], point-of-care diagnostics tools – for instance rapid testing for biomarkers, portable electrocardiography and bedside ultrasound –should be used to facilitate risk stratification, detection of co-morbidities and identification of complications such has heart failure and kidney disease. In addition, clinical protocols must be made available to allow immediate treatment of those at high risk of target organ damage. Moreover, despite recognizing that ED are not the ideal setting for patient health education, counseling on healthy lifestyle, risk-free behavior and adherence to therapy to selected populations should probably be considered in such setting, to avoid loss of opportunities to prevent complications, to reduce hypertension-related morbidity and mortality, as well as to support continuity of treatment after diagnosis. The use of preventive medicine health workers - who are currently involved in maternal and child health disease prevention – would probably be an important step towards better control of hypertension, if these professionals are trained to provide counseling and non-pharmacological therapy inside the health facility.

Inadequate supply of medicines is a major determinant for inadequate anti-hypertensive treatment and catastrophic spending in poor households and may (at least partially) explain the low levels of control in Mozambique [4**,** 6**,** 22]. Access to public hospitals is virtually free; patients pay $0.02 at entry points for all procedures within the health facility and $0.08 per full prescription, the total amount charged corresponding to 0.1% of the country’s minimum wage [11]. However, out-of-pocket expenditure for continuum of care in hypertension may be prohibitively expensive for the poorest households, as communities have high levels of poverty and informal employment, and almost no access to affordable private health insurance mechanisms. In Bangladesh, NCD-afflicted families allocate a greater share of household expenditures for medical care than households without NCDs and have almost seven times higher probability of incurring catastrophic medical expenditure, as well of selling assets or borrowing from informal sources to finance treatment cost [23]. In Mozambique, a considerable proportion of the household budget for families living on the national minimum wage is used to buy any anti-hypertensive, mainly if the patient needs more than one hypertensive medicine. Medical therapy entails large out-of-pocket expenditures and increases the likelihood of household impoverishment as shown in Kyrgyzstan, where households with an hypertensive patients had significantly higher total expenditure on health and drug therapy, thus being more prone to catastrophic health spending [22]. Similarly, it has been shown that patient costs associated with obtaining care for hypertension in public health care facilities in Kenya include substantial direct and indirect costs, as well as high rates of catastrophic costs [24]. Therefore, systems to assess the unmet needs of anti-hypertensive drugs, improve the whole supply chain in the public sector and financial risk protection for patients from these poor communities are needed.

Health System related factors impact on the capacity to control blood pressure [25**–**27] In African countries Health Systems are primarily oriented to managing infectious diseases, and therefore health professionals are unprepared to deal with NCD, given the scarcity of resources in health facilities [28]. The strategy of task-shifting - defined as the rational distribution of health care duties from physicians to non-physicians health care providers - is one of the effective approaches that has been used to address lack of human resources in Africa **[**29**]**, including for management of HBP [30**,** 31]. Considering the experience of Mozambique’s NHS in task shifting in obstetrics, surgery and mental health [20**,** 32], we strongly believe that targeted context-specific changes to the Health System in Mozambique can also be done to allow task-shifting for the diagnosis and management of hypertensive patients to occur, in order to improve rates of control and outcomes.

*Limitations:* Despite the acknowledging that the “snapshot” taken during one month cannot be fully representative of the situation, we believe that this description of hypertension care in ED constitutes an initial step towards addressing poor outcomes and understanding the organizational changes needed to improve care in our setting. Moreover, this model can be ameliorated and replicated for assessment of health care for other risk factors and NCD, thus supporting priority setting and selection of the most efficient interventions and health services changes that can be done to create context-tailored NCD clinics in Africa.

## Conclusions

Critical gaps in health facility readiness to address arterial hypertension seen in ED were uncovered in this highly prevalent low-income urban setting, including lack of clinical protocols, insufficient availability of diagnostics and essential medicines, as well as low affordability of the families to guarantee continuum of care. Our results show opportunities for improvement at the provider’s level, particularly of the organizational and managerial processes. Innovative financing mechanisms for the health system are needed to support the poorest, improve the rates of hypertension control and prevent poor outcomes.

## Data Availability

The datasets used and/or analyzed during the current study are available from the corresponding author on reasonable request.

## Abbreviations

AIDS: Acquired Immunodeficiency Syndrome
ED: Emergency Department
HAI: Health Alliance International
HbA1c: Glycated hemoglobin
HBP: High Blood Pressure
HGM: Mavalane General Hospital (Hospital Geral de Mavalane)
HIV: Human Immunodeficiency Virus
INS: National Health Institute (Instituto Nacional de Saúde)
NCD: Non-Communicable Diseases
NHS: National Health Service
WHO: World Health Organization
